# Notes and News

**Published:** 1899-01-07

**Authors:** 


### NOTES AND NEWS.

(COLLECTED AND COMMUNICATED.)

THE Prince of Wales has appointed Sir James Reid Physician in Ordinary to His Royal Highness, in succession to the late Sir William Jenner.

MR. CHARLES HEIDSIECK has presented 200 dozen bottles of champagne to the Prince of Wales's Fund, for use in the London hospitals.

INFORMATION has been received from St. Petersburg that the plague has broken out in Mongolia, where terribly insanitary condition prevails, coupled with great distress amongst the people.

SIR SQUIRE BANCROFT will tell the story of Charles Dickens' "Christmas Carol" at St. Martin's Town Hall, on Thursday afternoon, January 12th, at three o'clock, in aid of the Royal London Ophthalmic Hospital. The Right Hon. Sir John Lubbock, Bart., M.P., F.R.S. (president of the hospital) will preside. Tickets can be obtained from the secretary, Royal London Ophthalmic Hospital, Moorfields, E.C.

THE grants made, and the conditions attached to some of them, by the Council of the Prince of Wales's Hospital Fund for London have given very general satisfaction not only to the public but to the hospital authorities as a whole. It is instructive, therefore, to notice that a letter signed "A Hospital Secretary" has been published by the *Daily News* and the *Daily Chronicle*, the authorship of which is not far to seek. If the editors who published this letter would refer to the list of awards by the Prince's Fund and ask the honorary secretaries to confidentially show them the report of the Distribution Committee they would not, we fancy, give this correspondent, who wisely conceals his name, further space in their columns. The correspondent's animus is shown by the fact that "A Hospital Secretary" attacks the Prince's Fund because it advertised for applications, and states that the Sunday and other Funds have never taken this course. This is not correct, for the Hospital Sunday Fund has regularly advertised each year for applications since the commencement of that Fund. His other statements and figures are equally inaccurate and unreliable.

WITH the new year the *English Illustrated Magazine* commences a fresh series, in which, among other features, we notice the introduction of a number of coloured pictures in each number. *Science Gossip*, which has removed to new offices at 110, Strand, is another magazine which starts the year with renewed vigour.

AMONGST the munificent bequests to charity made by the late Baron Ferdinand de Rothschild is £100,000 to the Evelina Hospital for Children, founded in memory of the late Baroness. The Brompton Hospital for Consumption and St. George's Hospital each receive £1,000; whilst the Seamen's Hospital, in common with other seamen's charities, receives a handsome sum.

THE necessity of providing for the large class of epileptics who at present are unsuitably housed in the asylums appears at last to be recognised. The Asylums Committee of the County Council have recommended to the Council that a working colony should be established on the Horton Manor estate, an area of some 127 acres. Outdoor labour with suitable supervision and accommodation would be provided. Hitherto the only accommodation for epileptics has been provided through private generosity, and the success which has attended the schemes already established has encouraged the County Council to consider adopting like arrangements for the epileptics under their charge.

AMONG the honours granted by the Queen on the occasion of the New Year we note that Sir Henry Thompson, F.R.C.S., is made a baronet. Knighthood has been conferred upon Dr. Hermann Weber, well known within the profession for his researches on many subjects, but perhaps best known to the public as an authority on watering-places and on the hygienic and climatic treatment of phthisis. Knighthood has also been conferred upon John Furley, Esq., so well known in connection with ambulance work. Sir Hugh Owen, K.C.B., receives the well-deserved honour of promotion to be G.C.B. Sir Charles Cameron, M.D., so well known as medical officer of health for Dublin, and for his researches in regard to the relation between typhoid fever and conditions of the soil, is made a C.B. T. E. Macpherson, Esq., M.D., and R. N. Moffat, Esq., M.D., officers of the Uganda Administration, are made Companions of the Order of St. Michael and St. George. Colonel William Pleace Warburton, M.D., and Colonel David Sinclair, M.B., of the Indian Medical Service, have been made Companions of the Most Exalted Order of the Star of India. Lieutenant-Colonel Henry Kellock McKay, and Major Winthropp Benjamin Browning, of the Indian Medical Service, have been made Companions of the Most Eminent Order of the Indian Empire. Knighthood has been conferred upon Dr. Plunkett O'Farrell, Commissioner of Control and Inspector of Lunatic Asylums in Ireland. The Queen has been pleased to approve the following appointments in the Order of the Hospital of St. John of Jerusalem in England, in recognition of the distinguished services rendered by the persons named during the plague epidemic in India: To be enrolled Honorary Associates of the Order—Captain John Lloyd Thomas Jones, Indian Medical Service; Captain William Ernest Jennings, Indian Medical Service; Captain Arthur Frederick William King, Indian Medical Service; and Lieutenant William James Niblock, Indian Medical Service.

